# Supplementary material for: Significant Impact of Coffee Consumption on MR-Based Measures of Cardiac Function in a Population-Based Cohort Study without Manifest Cardiovascular Disease
Source: Nutrients. 2021 Apr 13;13(4):1275. doi: 10.3390/nu13041275 (PMC8069927; doi:10.3390/nu13041275)
Supplement: Supplementary file 1 [file nutrients-13-01275-s001.zip › Suppl/Table S1.docx]

**Table S1: MRI based cardiac function comparing low, middle and high coffee-consumption**

| **Coffee intake**  **(g/day)** | **Middle Tertile**  **(383.6-458.4)** | **High Tertile**  **(459.0-699.2)** |
| --- | --- | --- |
|  | *β (95%CI)* | *β (95%CI)* |
| Early diastolic filling rate (ml/s) | -4.99 (-34.6; 24.62) | 1.20 (-29.21; 31.61) |
| Late diastolic filling rate (ml/s) | 33.43 (-3.27; 70.12) | 28.58 (-9.11; 66.26) |
| End-diastolic volume (ml/m^2^) | -0.26 (-4.20; 3.68) | 0.88 (-3.16; 4.93) |
| End-systolic volume (ml/m^2^) | -1.34 (-3.55; 0.88) | -1.26 (-3.53; 1.02) |
| Stroke volume (ml/m^2^) | 1.02 (-1.55; 3.59) | 2.14 (-0.50; 4.79) |
| Ejection fraction (%) | 1.89 (-0.26; 4.04) | **2.22 (0.01; 4.43)*** |
| Peak ejection rate (ml/s) | -9.55 (-45.19; 26.09) | 2.92 (-33.68; 39.53) |
| Myocardial mass (g/m^2^) | 2.57 (-0.69; 5.82) | 1.91 (-1.43; 5.25) |

Reference group: low tertile of coffee intake (39.5-383.2 g/day)). β-coefficients are from linear regression models adjusted for age and sex, smoking, hypertension, diabetes, LDL, triglycerides and alcohol consumption, n=300, *p<0.05; **p<0.01.
