# Supplementary figures and images for: Significant Impact of Coffee Consumption on MR-Based Measures of Cardiac Function in a Population-Based Cohort Study without Manifest Cardiovascular Disease
Source: Nutrients. 2021 Apr 13;13(4):1275. doi: 10.3390/nu13041275 (PMC8069927; doi:10.3390/nu13041275)

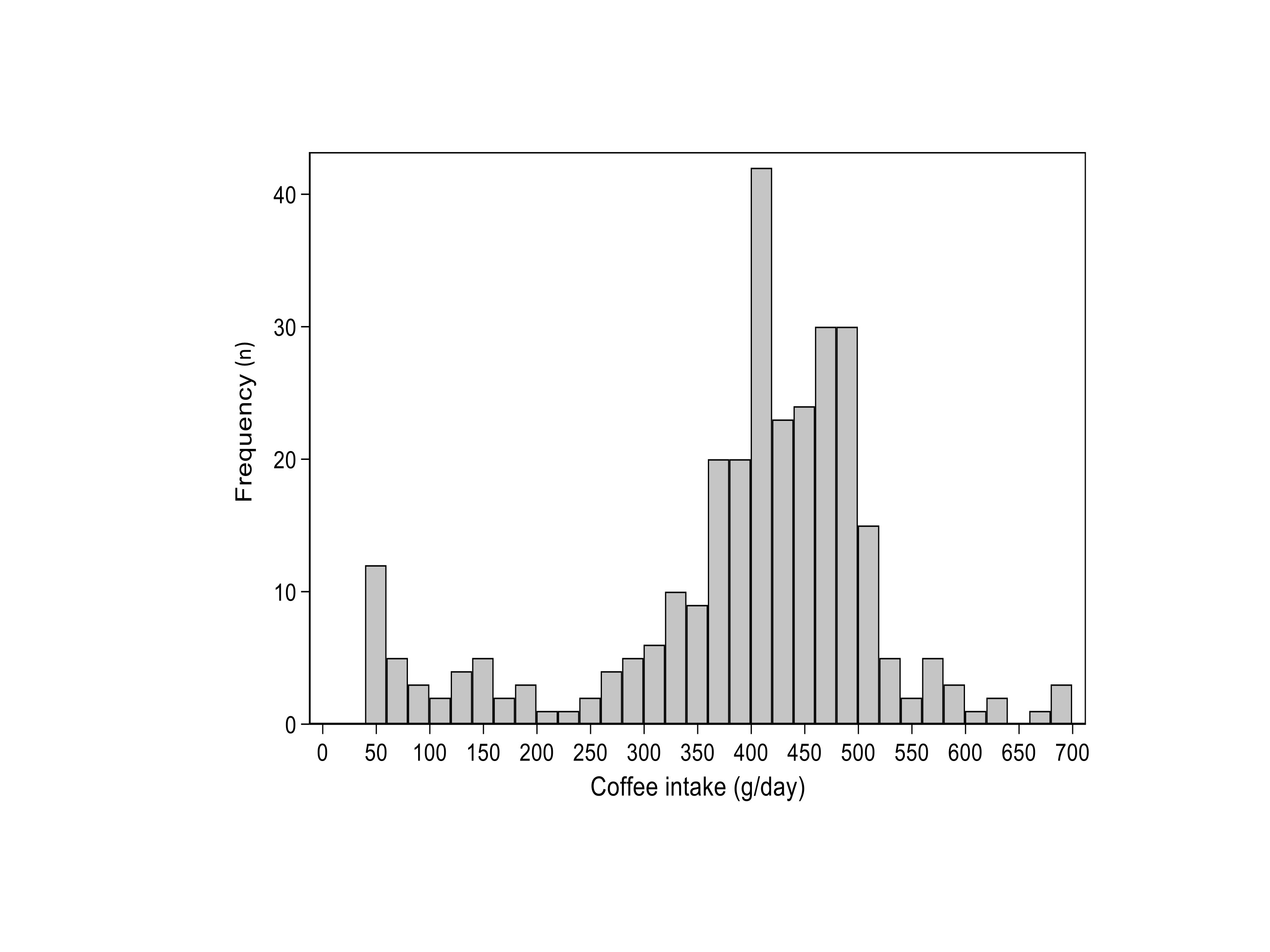

Supplement: Supplementary file 1 [file nutrients-13-01275-s001.zip › Suppl/Figure S1.jpg]
